# Trends in the Hydrogen−Deuterium Exchange at the Carbon Centers. Preparation of Internal Standards for Quantitative Analysis by LC-MS

**DOI:** 10.3390/molecules26102989

**Published:** 2021-05-18

**Authors:** Paulina Grocholska, Remigiusz Bąchor

**Affiliations:** Faculty of Chemistry, University of Wrocław, F. Joliot-Curie 14, 50-383 Wrocław, Poland; paulina.grocholska@chem.uni.wroc.pl

**Keywords:** hydrogen−deuterium exchange, liquid chromatography-mass spectrometry, *N*-methylated amino acids, quantitative LC-MS analysis

## Abstract

The application of internal standards in quantitative and qualitative bioanalysis is a commonly used procedure. They are usually isotopically labeled analogs of the analyte, used in quantitative LC-MS analysis. Usually, ^2^H, ^13^C, ^15^N and ^18^O isotopes are used. The synthesis of deuterated isotopologues is relatively inexpensive, however, due to the isotopic effect of deuterium and the lack of isotopologue co-elution, usually they are not considered as good internal standards for LC-MS quantification. On the other hand, the preparation of ^13^C, ^15^N and ^18^O containing standards of drugs and their metabolites requires a complicated multistep de novo synthesis, starting from the isotopically labeled substrates, which are usually expensive. Therefore, there is a strong need for the development of low-cost methods for isotope-labeled standard preparations for quantitative analysis by LC-MS. The presented review concentrates on the preparation of deuterium-labeled standards by hydrogen−deuterium exchange reactions at the carbon centers. Recent advances in the development of the methods of isotopologues preparation and their application in quantitative analysis by LC-MS are evaluated.

## 1. Introduction

Hydrogen−deuterium exchange (HDX) is a process involving the substitution of a hydrogen atom by a deuterium atom in a molecule of a chemical compound, in the presence of a deuterating agent, e.g., deuterium oxide (D_2_O), or another source of dissociating deuterons [1,2]. Labile hydrogens in the backbone and side-chain functional groups of chemical compounds undergo exchange with protons of the solvent within a few minutes [1]. Due to the basic mechanism of the H/D exchange reaction, including acid-base catalysis, the degree of the reaction depends strongly on the pH of the solution [2]. The decimal logarithm of the reaction rate constant versus pH curve takes a V-shape with a characteristic minimum between pH 2 and 3, where the average half-time of the exchange at 0° C is tens of minutes. The rate of H/D exchange increases significantly with increasing pH. The rate of the H/D exchange reaction depends also on the acidity of the hydrogens bounded with heteroatoms, which is a consequence of the electronegativity differences between these atoms. For this reason, hydrogens from carboxyl or hydroxyl groups undergo the H/D exchange reaction much more easily than amide ones. The role of the inductive effects of the individual functional groups present in the vicinity of the hydrogen undergoing H/D exchange, and of steric hindrance, hindering the access of the acid or basic catalyst to the exchangeable proton is also described [1,2].

The influence of the molecular structure of the compound on the isotope exchange has also been observed, especially in the case of peptides and proteins. It was found that amide hydrogens participating in intramolecular hydrogen bonds were much less susceptible to isotopic exchange reactions. Therefore, the H/D exchange was found as a tool in the conformation analysis of biologically active compounds. Additionally, the isotope exchange reactions enabled the analysis of the mechanisms of chemical processes [3]. The compelling advantage of mass spectrometric analysis of the H/D exchange of peptides and proteins is the high sensitivity of the method, the low concentrations of the analyte which can be used and the ability to simultaneously analyze individual components of the complex mixtures [4].

**Figure 1:**
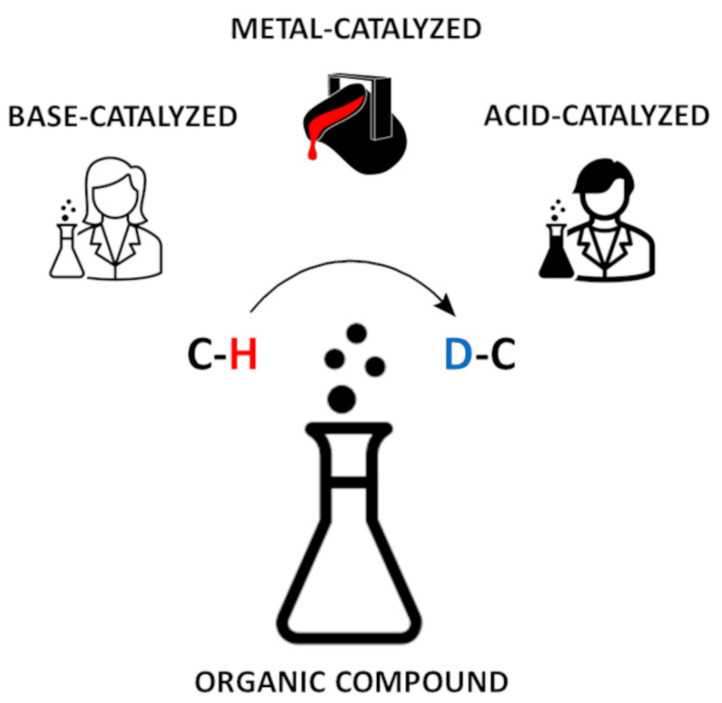
Hydrogen−deuterium exchange catalyzed by base, acid or metal catalyst.

In contrast to the hydrogens attached to heteroatoms, the hydrogen atoms bound to the carbon atoms are usually not exchangeable, however, the specific reaction conditions, including pH-dependent and metal-dependent catalysis, may promote the replacement of carbon-attached protons by deuterons (Figure 1) [5,6,7,8]. Such compounds may serve as internal standards in quantitative LC-MS analysis. Therefore, the exchange of hydrogen attached to the carbon by its heavier isotopes are of interest for mechanistic, product-orientated research and quantitative analysis [3]. The existing methods of isotope exchange on the α-carbon atoms of amino acids are expensive and time-consuming, because they require multistep de novo synthesis with the application of isotopically labeled substrates. Usually preparation of such deuterated derivatives by hydrogen−deuterium exchange is easier and more cost effective than by classical de novo synthesis [7]. Therefore, the development of new, ‘gentle’ methods of isotope exchange on the α-C carbon atoms of amino acid residues in peptides seems to be an important issue. At present, isotopically labeled compounds at the carbon atoms are of interest, especially due to their application as internal standards in quantitative mass spectrometry in the analysis of fragmentation mechanisms.

Liquid chromatography-mass spectrometry (LC-MS) quantification frequently is performed in the presence of isotopically labeled standards which in most cases have to be synthesized de novo [9,10]. There are certain requirements for these standards: isotopologues should be characterized by identical chromatographic behavior, the mass difference between isotopologues should be at least 2 Daltons and the introduced isotopes cannot undergo back exchange during LC-MS separation conditions [11]. Quantification is performed by MS analysis, by comparing the extracted ion chromatograms (peak area) of the isotope-labeled and nonlabeled compounds. Although various stable isotope-labeled quantification reagents have been described containing ^2^H, ^13^C, ^15^N and ^18^O isotopes, due to their complicated and expensive chemical synthesis, there is still a strong need to develop a method of preparation of new isotopically-labeled standards. Usually, the preparation of deuterated standards is relatively inexpensive, however, the possibility of isotope effect affecting their co-elution during LC-MS would limit their possible application in LC-MS quantification [12,13]. It was found that the isotope effect on the chromatographic behavior of deuterated and nondeuterated isotopologues depends on the number and place of the introduced deuterons [14,15]. Additionally, deuterium-labeled compounds cannot be used for in vivo studies due to the possible loss of deuterium or different metabolism pathways. Therefore, there is a strong need for low-cost methods for isotope-labeled standard preparation for quantitative analysis by LC-MS. Although, the incorporation of deuterium into the target molecules may present some drawbacks, nevertheless the most important advantage of such a procedure is its low cost and simplicity of preparation.

In this review, we present the methods of deuterium incorporation into the molecules of compounds by exchange reactions and the possible application of deuterated standards in quantitative analysis by LC-MS.

## 2. Hydrogen−Deuterium Exchange at Carbon Centers

### 2.1. Acid- and Base-Catalyzed HDX

The pH-dependent hydrogen−deuterium exchange reactions are the first reported methods in the presented field [16]. The acid and base-catalyzed HDX process involves enolization which makes the H/D exchange at activated carbon centers possible in the presence of a source of deuterium, including deuterated Brønsted acids or bases. The back exchange of introduced deuterons is of course possible, therefore further deactivation of the analyzed compound is required.

In most cases, acid-catalyzed H/D-exchange reactions are used to incorporate the deuterium into the aromatic molecules. In these cases, strong deuterated Brønsted or Lewis acids, in the presence of a deuterium source are commonly used. The application of Lewis acids, including AlBr_3_ or MoCl_5_ is mostly restricted to the nonpolar arenes. In acid-catalyzed HDX, the incorporation of the ^2^H isotope to the aromatic compounds exhibits limited regioselectivity. The effect of the aromatic ring substituents on the deuteration regioselectivity was analyzed in the acid-catalyzed HDX on ferrocenes. It was found that the electrophilic aromatic deuteration of the cyclopentadienyl rings was favored by alkyl groups whereas enolization of the carbonyl group in ketones led to the selective and complete H−D exchange of all three hydrogens of the acyl residue [17].

Base-catalyzed HDX is also a facile method for deuterium incorporation by means of keto–enol equilibria. Due to the higher acidity of carbon-bound hydrogen atoms in carbonyl compounds, including ketones [18], aldehydes [19], esters [20] and carboxylic acids [21], they undergo H/D exchange with high selectivity (>90% D) and yield. The γ hydrogens in α,β-unsaturated carbonyl compounds are also able to exchange through conjugation, as presented on the steroid framework of androstenedione, testosterone, and cortisone [22].

**Figure 2:**
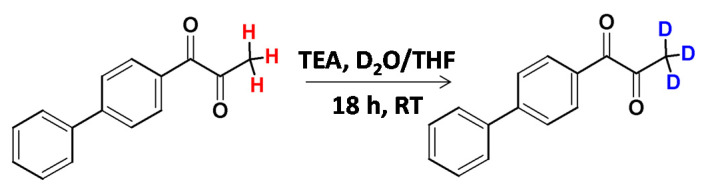
Deuteration of diketone presented by Berthelette and co-workers [23].

The deuteration of the methyl group in aryl methyl ketones and aryl methyl sulfones under basic conditions was presented by Berthelette and Scheigetz [23]. It was found that the reaction efficiency and rate depended on the base, the substrate and the solvent nature. Whereas 1,8-diazabicyclo[5.4.0]undec-7-ene (DBU) was found as a base allowing high deuteration efficiency, *N,N,N*-triethylamine (TEA) was less effective in the corresponding HDX processes (Figure 2). TEA was found as a base allowing methyl group deuteration in the base-sensitive ketones without decomposition.

The base-catalyzed HDX reaction is a simple method for the acidic hydrogen exchange for deuterium by keto-enol equilibria [16]. The carbon-bound acidic hydrogens in carbonyl compounds, including *N*-substituted acetamides or diketopiperazines are usually highly exchanged [5]. The presented H/D exchange involved the application of acetone-d_6_, *N*,*N*,*N*-triethylamine (TEA) or a stronger base in the form of diazabicycloundec-7-ene (DBU) and incubation at higher temperatures (35 °C to 50 °C). The acidity of α-carbon hydrogens of amino acids and peptides is of interest because the corresponding enolates play an important role in nonenzymatic racemization during peptide synthesis and enzyme-catalyzed racemization in different biochemical transformations [5,24]. Till now only a few quantitative investigations on their stability in water have been published. Their formation rate constants have been analyzed and determined in studies of α-hydrogen exchange or racemization reactions of amino acids and peptides at high temperatures [25,26] for the development of base-catalyzed methods for the preparation of α-C-deuterated amino acids is an important research task [8,27]. Generally, synthetic methods are based on the glycine derivatives application, which are subjected to a basic HDX and the stereoselective insertion of the desired side chain.

The acidity of α-C hydrogens of various amino acids and their derivatives have been extensively investigated. Ho et al. informed that α-C hydrogens in *N*-methylated analogs of cyclic dipeptides are more acidic than those in nonmethylated compounds [5,28]. It was also confirmed that the substitution of the amino group with electron-withdrawing substituents, such as the acetyl group, facilitated the HDX of α-C hydrogens. It was found by Rios and co-workers that the acidity of the α-C hydrogens depended on the ionization state of the amino acid, additionally the exhaustive methylation of α amino group also affected the pKa of the presented hydrogens [29,30]. Up to now, several different compounds containing *N*-methylated amino acids in their chemical structure have been described. These compounds belongs to the group of important drugs and natural tissue metabolites or substances commonly used in industry and households. In many cases there is a need to quantify them.

**Figure 3:**
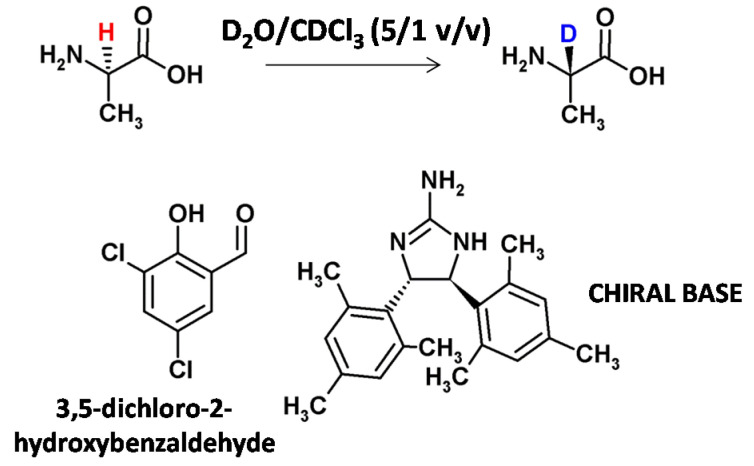
Catalytic stereoselective deuteration of L-alanine to deuterated D-alanine in the presence of 3,5-dichloro-2-hydroxybenzaldehyde and a chiral base [31].

Moozeh and co-workers presented the stereoinversion of L-alanine to α-C deuterated D-alanine by base catalysis [31]. In the presence of salicylaldehyde and a chiral base, 87% deuterium incorporation at α-C of L-alanine and an inversion to D-alanine, was obtained. The enantiomeric excess (ee) was 67% (Figure 3). The developed method was also successfully applied for the deuteration of another 11 natural amino acids (threonine, tryptophan, phenylalanine, methionine, glutamic acid, glycine, glutamine, asparagine, serine, lysine and leucine). Stereoinversion for the presented examples was not reported.

**Figure 4:**
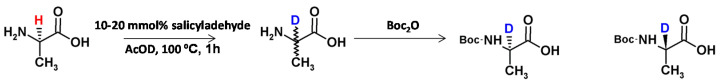
Deuteration of amino acids under acidic conditions [16].

Mitulovi and co-workers [32] presented the method of acid-catalyzed deuteration of α-amino acids (i.e., alanine, leucine, phenylalanine). In the presence of [D1]acetic acid (excess) and catalytic amounts of aldehyde, the reaction is characterized by good yield and the deuterium incorporation at the level of 95% via the corresponding Schiff’s base formation (Figure 4). The obtained compound was converted into the tertbutoxycarbonyl (Boc) protected derivative and the resulting enantiomeric mixture was separated by preparative high-performance liquid chromatography on a chiral stationary phase.

The methods for the preparation of enantiomerically pure α-C-deuterated amino acids in the presence of a base are based on the application of glycine or its derivatives, which are subjected to a basic HDX. Finally, the side chain is inserted stereoselectively with the aid of chiral auxiliaries [6,7,8].

**Figure 5:**
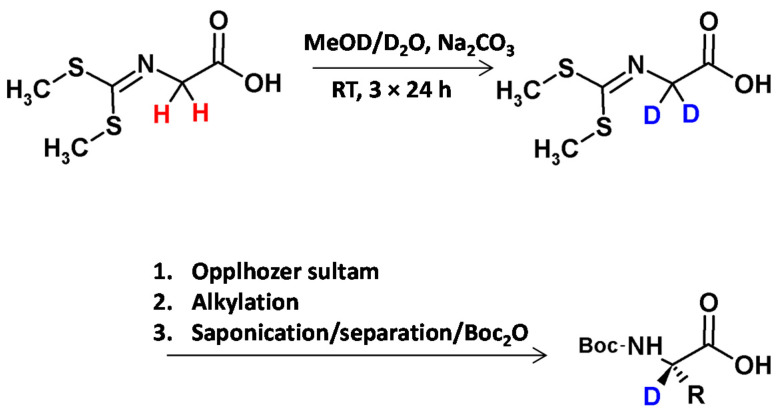
Stereoselective synthesis of deuterated amino acids [16].

Lankiewicz and co-workers [7], described the method of the preparation of deuterated glycine derivative in the mixture of MeOD/D_2_O and the presence of catalytic amounts of Na_2_CO_3_ as a base. After three reaction steps of reaction the obtained derivative was characterized by a deuterium content greater than 98%. Then the reaction with the Oppolzer sultam provide an intermediate for the subsequent stereoselective alkylation [33]. After removal of the auxiliary, the chiral Boc-protected amino acid (glycine, alanine, leucine, phenylalanine, O-benzyltyrosine) was isolated almost enantiomerically pure (>99% ee) in high yield (Figure 5).

**Figure 6:**
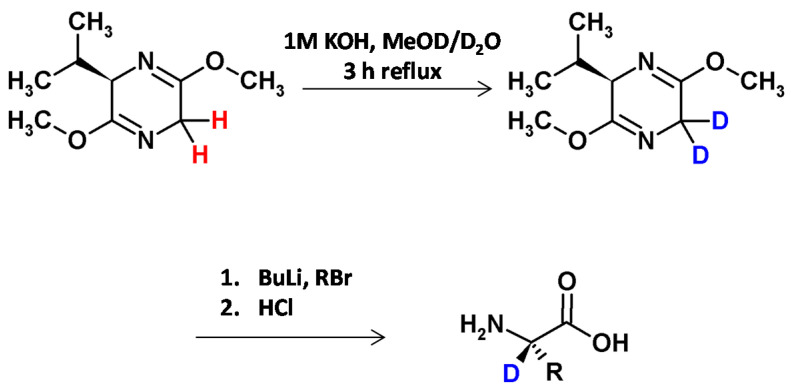
Schӧllkopf bislactim ether application in the preparation of deuterated amino acids [16].

Rose and et al. [8], inspired by the bislactim ether method developed by Schöllkopf and co-workers [34], described a base-catalyzed method of C6-position deuteration of the dihydropyrazine in boiling mixture of MeOD/D_2_O (Figure 6). No hydrogen−deuterium exchange was observed at the C3-position due to the steric hindrance of the isopropyl group in the transition state. The obtained [6-D2]isotopologue was stereoselectively alkylated at the C6-position, thereby giving access to a series of α-C-deuterated amino acids (serine, phenylalanine, allylglycine, aspartic acid) in good yields, high degrees of deuteration and enantiomeric excesses (>95%) [8].

**Figure 7:**
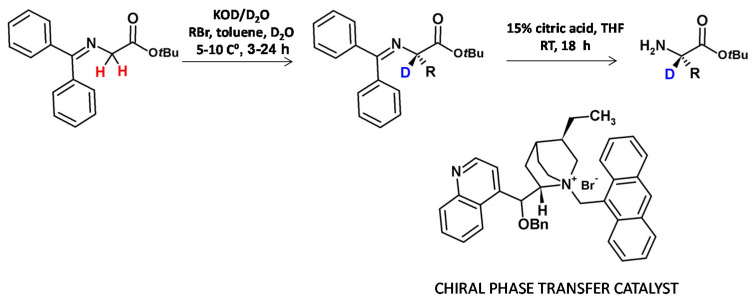
Enantioselective deuteration of amino acids in the presence of chiral phase transfer catalyst [16].

A further enantioselective synthesis of α-C-deuterated (Figure 7) proceeds by asymmetric alkylation of the activated glycine in the presence of the chiral phase-transfer catalyst. The HDX in the presence of KOD in D_2_O and the introduction of the side chain were performed in a single reaction step. After imine hydrolysis the amino acid *tert*-butyl esters were isolated in good yields and with high deuterium incorporation of more than 90% [6].

In addition to hydrogen−deuterium exchange based on the keto-enol tautomerism, the deuterolysis of an organometallic compound is also the chemical tool used for the synthesis of deuterated derivatives. In this reaction, the intermediate organometallic compound is formed by deprotonation in the presence of strong bases (i.e., Grignard reagent or alkyl–lithium compound), and subsequently deuterated with electrophiles in the form of D_2_O, MeOD, or AcOD; which formally correspond to the H/D exchange [35]. Using this approach complete ortho-deuteration of aromatic amides and aromatic carbamates was achieved. Moreover, due to the large kinetic isotope effect (KIE), incorporated deuterium served as a protecting group for the carbon center, allowing the control of the regioselectivity of the subsequent lithiation.

Hydrogen−deuterium exchange reactions can also be performed without the addition of acids and bases. Such transformations are characteristic for the acidic CH centers which may be deuterated simply by the incubation of compound in deuterium oxide. The autoprotolysis equilibrium of D_2_O, makes it possible to act as either an acid or a base. For example, the synthesis of [1,1,3,3-D4]2-indanone was achieved by the heating of the compound of interest in the D_2_O [36]. Other reactions, depending on the compound, which were suspected to be HDX, required sometimes drastic reaction conditions which cannot be applied for most organic molecules.

A simple strategy was presented by Pacchioni et al. [37]. It was found that the formation of *N,N,N*-trinitroso derivative of the 1,4,7-triazacyclononane made the α-methylene hydrogen atoms more acidic allowing HDX in the presence of base.

A variant of acid-catalyzed HDX, which uses only D_2_O during the deuteration process and is accelerated by microwave irradiation, was presented by Barthez et al. [38]. The developed strategy was also successfully applied to aminopyridine derivative preparation [39]. In order to avoid any proton sources, the labile hydrogen atoms bound to the nitrogen were exchanged to deuterons in the presence of D_2_O. The applied strategy allowed complete deuteration within a few minutes and a high deuterium content at the *ortho* and *para* positions in the amino group.

Very recently, the application of microwaves in HDX processes significantly increased, especially due to the higher degree of deuteration, shorter reaction times as compared to the classical heating conditions. Based on this technique several MS standards of bleomycin A2 for quantitative MS analysis were successfully prepared in D_2_O after a two-minute heating at 165 °C [40]. Additionally, some physicochemical reports have been published in which the kinetics of noncatalyzed HDX and energetic investigations were described [41].

It was previously reported that the base-catalyzed hydrogen−deuterium exchange at the carbon centers of aldehydes and ketones, thioesters and oxygen esters or amides occurred via a stepwise mechanism involving the enolate intermediate formation when the enolate was sufficiently stable to exist for the time of a bond vibration [30].

*N*-methylglycine, also called sarcosine, represents a natural, achiral compound with a methylated amino group which plays an important role in biological systems [42]. This amino acid residue is present in cyclosporine A, a cyclic nonribosomal peptide, commonly used as immunosuppressant [43]. Methods for the quantitative analysis of cyclosporine A and its metabolites have been developed [44] however, due to the necessity of preparation of its isotopically labeled standards for MS quantification, the costs of such analysis were very high. Joining the mainstream of the base-catalyzed HDX, previously, we developed several methods of preparation of deuterated standards of compounds containing *N*-substituted glycine derivative in their chemical structure, including denatonium benzoate, peptomers, cyclosporin A and creatinine. Additionally, the applicability of the obtained deuterated standards were tested in the quantitative analysis of these compounds by LC-MS.

**Figure 8:**
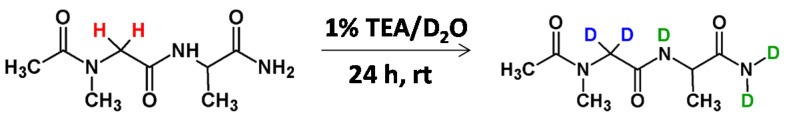
Schematic presentation of H/D exchange reaction at the α carbon in *N*-methylglycine moiety [45]. TEA-*N,N,N*-triethylamine.

In our previous work, the base-catalyzed HDX of α-C hydrogens in sarcosine residue and specific hydrogen scrambling in such peptides were investigated [45]. We found the unusual hydrogen−deuterium exchange at the α-carbon in *N*-methy- and *N*-benzylglycine residues in the presence of 1% solution of *N,N,N*-triethylamine in D_2_O (Figure 8). We found that the observed HDX proceeded at a much slower rate as compared to the hydrogen−deuterium exchange of hydrogens present in amines or amides. Moreover, we observed the hydrogen scrambling during the collision-induced dissociation experiment which suggested the lability of such hydrogens. The presented work opened a wider possibility of application of the presented HDX reaction in peptide chemistry and mass spectrometry.

**Figure 9:**
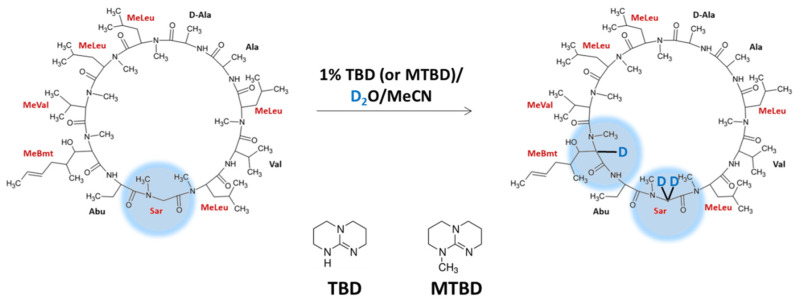
Preparation of deuterated analogs of cyclosporin A as described by Bąchor et al. [46].

Our investigation on compounds containing sarcosine residue revealed its presence in cyclosporin A molecule. The analysis of the possibility of the α-C deuteration of cyclosporine A (CsA) in *N*-methylated amino acid residues was performed [46]. The proposed reaction is based on the method previously reported by us [45], proceeds under basic conditions in the presence of TBD (1,5,7-triazabicyclo[4.4.0]dec-5-ene) or MTBD (7-methyl-1,5,7-triazabicyclo[4.4.0]dec-5-ene) at pH 13.4 (Figure 9). The obtained results revealed that there is a possibility of three deuteron incorporation, two at the α-C of *N*-methylglycine and one at the α-C of 2-*N*-methyl-(R)-((E)-2-butenyl)-4-methyl-L-threonine (MeBmt) residue. The prepared isotopologues were stable (did not undergo back exchange) under neutral and acidic conditions. Additionally, the deuterated and nondeuterated derivatives revealed co-elution, which make their application for the quantitative analysis by using isotope dilution strategy possible. The developed strategy of the CsA deuteration is rapid, cost-efficient and does not require special reaction conditions, other reagents or further purification [46].

The synthesis of the α-C deuterium-labeled *N*-substituted glycine residues in peptomers—oligomers composed of both α-amino acids and *N*-substituted glycine monomers—at basic conditions at room temperature was also analyzed by Bąchor et al. [47]. The developed method covered the deuterium labeling of peptomers at the α-C atom of *N*-substituted glycine residues by using simple HDX. The proposed labeling procedure is easy, inexpensive, and does not require any derivatization reagents or further purification. The introduced deuterons do not undergo a back-exchange under neutral and acidic conditions during LC-MS separation. The LC-MS analysis of an isotopologue mixture showed their co-elution. Therefore the developed strategy may be applied in the quantitative isotope dilution analysis of peptoids and other derivatives of *N*-substituted glycines.

**Figure 10:**
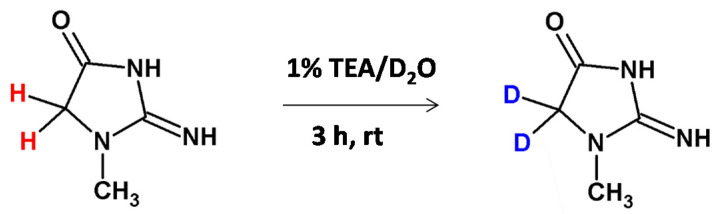
H/D exchange at the carbon center of creatinine molecule [48].

Very recently we developed a method of deuterium-labeled standard preparation of creatinine, a breakdown product of creatine phosphate in muscle and a molecular biomarker of renal function [48]. The *N*-methylated glycine moiety was also presented within the creatinine molecule. The performed investigation allowed the doubly deuterated Cre analogue to be obtained, even after 60 min incubation in 1% TEA/D_2_O solution at room temperature (Figure 10). We found that the introduced deuterons were stable under acidic and neutral conditions and any back exchange was observed. The obtained results suggest that the obtained deuterated Cre analogue may serve as a good internal standard for quantitative analysis by ESI-MS by using the isotope dilution method. The proposed methodology is a new, inexpensive and simple way for creatinine quantification. Additionally the performed quantification in the presence of the obtained deuterated Cre standard correlates with the Jaffe test method.

**Figure 11:**
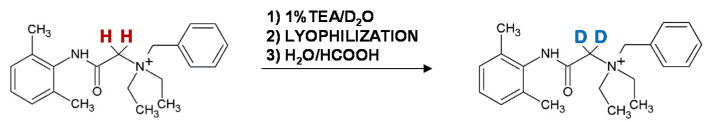
Preparation of deuterated Bitrex standard for quantitative analysis by mass spectrometry [49].

In 2015, the method of denatonium benzoate (Bitrex) deuteration via HDX of α-carbon hydrogens located in the CH_2_ group, situated between carbonyl carbon and quaternary nitrogen atom (Figure 11) [49]. The reaction proceeded at room temperature and the deuteration was completed after 1 h of sample incubation in 1% TEA/D_2_O mixture. We found that the introduced deuterons did not undergo back exchange under acidic and neutral conditions. We also found that the isotopologues—deuterated and nondeuterated—denatonium cation co-elute during the chromatographic separation. The applicability of the obtained deuterated denatonium cation as the internal standard for quantitative analysis of Bitrex was confirmed by the LC-MS analysis of various Bitrex-containing household products. The proposed strategy is a new and simple solution for sensitive Bitrex quantification by LC-MS method. We found that the presence of a quaternary nitrogen atom connected with the α-C atom facilitated the H/D exchange. Based on this observation, we focused our attention on the compounds containing quaternary ammonium groups in the form of *N*-substituted glycine derivatives. We reported the influence of the quaternary ammonium group on HDX at the α-C of sarcosine and *N*-methylalanine in peptides [50,51,52]. The significant acceleration of the HDX in sarcosine residue caused by the presence of a fixed charge tag was found. The effect depended on the distance between the sarcosine residue and the quaternary nitrogen atom. The deuterium atoms introduced at the α-C, did not undergo back-exchange under acidic aqueous solution. The tandem mass spectrometry analysis of the deuterated analogs of quaternary ammonium-tagged oligosarcosine peptides without mobile hydrogen showed the mobilization of the hydrogens localized at α-C atom of sarcosine residue.

It was presented previously that the racemization and hydrogen−deuterium exchange at the α-amino carbon atoms in dipeptides may proceed via the reversible diketopiperazine intermediate formation [53,54]. It was also assumed by Rios and co-workers that the exchange of hydrogens into deuterons at the α carbon atom in amino acid residues occurs via a stepwise mechanism catalyzed by DO^-^ anion where the enolate intermediate is formed [30]. The most important advantage of the acid or base catalyzed HDX is their simplicity of preparation, relatively low costs to perform, and mostly high efficiency. It should be also be pointed out that sometimes the hard reaction conditions, including high or low pH values, may lead to the compound decomposition.

### 2.2. Metal-Catalyzed HDX Adjacent to Oxygen Atom

**Figure 12:**
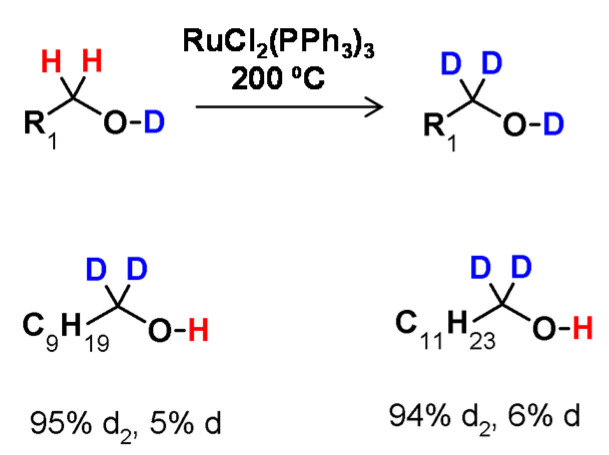
H/D exchange adjacent to oxygen atom, presented by Regen [55].

The first example of H/D exchange adjacent to oxygen was discovered in 1974 by Regen. In this study, the deuteration of 1-butanol at the α carbon atom was performed in the presence of a catalyst in the form of (tris(triphenylphosphine) ruthenium dichloride [RuCl_2_(PPh_3_)_3_] [55]. In the proposed method 1-butanol was incubated at 200 °C for one hour in the presence of a catalyst, which allowed the exchange of hydrogen at the α carbon position by deuterium bonded to the oxygen atom (Figure 12). The proposed reaction conditions were also applied to other deuterated alcohols at the α-C atom. The obtained results also revealed that the addition of D_2_O to the reaction mixture significantly increased the deuteration efficiency and that the degree of deuteration depended on the D_2_O/alcohol ratio.

**Figure 13:**
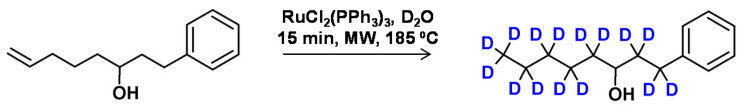
Ruthenium-mediated deuteration according to Ishibashi et al. [56].

Ishibashi et al. presented the efficient deuteration of compounds containing electron donors in the form of double bonds, hydroxyl groups, in the presence of ruthenium catalyst. It was found that alkenols were efficiently deuterated in D_2_O by the migration of the double bond and isomerization to ketones in the presence of ruthenium catalyst (Figure 13) [56]. It was found that primary alcohols were oxidated to aldehydes on the selective way in the presence of RuCl_2_(PPh_3_)_3_ catalyst which was hampered in the presence of small amounts of water. The reaction temperature around 150 °C in microwave synthesizer, allowed an efficient HDX at the α carbon atom of the primary alcohols with small epimerization observed in the case of chiral compounds. Under lower temperatures around 100 °C the epimerization was sufficiently suppressed [57].

In 2011, Bossi and co-authors presented a method for selective deuteration of alcohols at the α position in the presence of ruthenium and osmium pincer catalysts [58]. In the presence of isopropanol-d8 as a source of deuterium, Bossi was able to obtain high deuterium incorporation at the C1 carbon atom of primary and secondary alcohols, and within the case of secondary alcohols also the deuteration at the C2 position was observed. In 2013, Khaskin and Milstein proposed another ruthenium pincer catalyst allowing deuteration at the carbon centers in the presence of D_2_O and with a lower catalyst loading [59]. Other substrates, including secondary alcohols also presented the possibility for β deuteration, which was frequent, with deuterium incorporation up to 97%.

**Figure 14:**
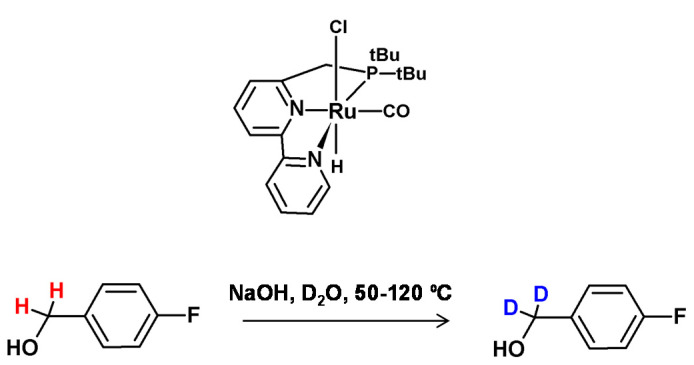
Khaskin’s work on alcohol deuteration catalyzed by ruthenium pincer catalyst [59].

In 2015, Bai et al. [60] proposed a selective α and β deuteration method of alcohols in the presence of D_2_O as a deuterating agent. The reaction optimization process revealed that octahedral ruthenium complexes with the amine ligand presented higher activity in HDX promotion (Figure 14). It was also found that the (η6-cymene) ruthenium complex allowed deuteration only at the β position. The mechanism of this process involves oxidation of the alcohol to an aldehyde followed by base-mediated β deuteration. The formed aldehyde is then reduced to an alcohol by means of the deuterated ruthenium complex which results in the formation of α,β-deuterated derivative.

**Figure 15:**
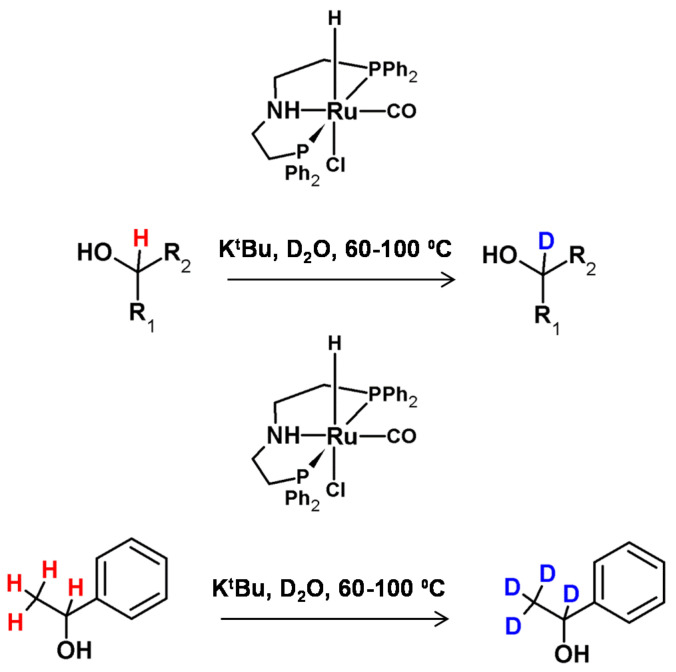
H/D exchange catalyzed by Ru-MACHO catalyst [61].

In the same year, Chatterje et al. presented the method of selective α and α,β deuteration of alcohols in the presence of a low-loading and commercially available ruthenium pincer catalyst (Ru-MACHO), the base in the form of potassium *tert*-butoxide and D_2_O as the deuterating agent (Figure 15) [61]. The proposed reaction condition optimization on aromatic benzylic alcohols revealed that mild heating (60–100 °C) was sufficient to obtain 95% of deuterium incorporation. It was also presented, that in this method linear primary alcohols were also able to undergo β deuteration at 10–20%, while in the case of secondary alcohols, deuteration at the β carbon was higher. The mechanism of this reaction involves the oxidation and reduction of the alcohol. Based on this, the authors proposed that the intermediate, in the form of ketones, was a more long-living species than the aldehydes and therefore, β deuteration proceeded at a higher rate in the case of secondary alcohols. What is interesting, in the case of diols, only the deuteration at the α position was observed.

Similarly to Khaskin [59], in 2016 Gauvin and co-workers focused their attention on the application of ruthenium pincer complexes in the transformation of alcohols to carboxylic acids. They performed the reaction in a closed vessel to displace the reaction equilibrium toward the substrate (alcohol), thus allowing deuteration in the presence of D_2_O [62]. They also decided to optimize the catalyst by switching from the Ru-MACHO to its analog containing cyclohexyl substituents, resulting in the higher activity and selectivity in α carbon deuteration.

Using a similar strategy to Regen’s method, Koch and Stuart found that there was a possibility for primary and secondary alcohol deuteration at the α carbon by refluxing the alcohol in the presence of D_2_O and Raney nickel as the catalyst [63]. The proposed strategy was successfully applied in the preparation of deuterated nonreducing carbohydrates. Additionally, it was proposed that the alcohol should undergo a redox process in the presence of Raney nickel as hydrogen-transfer catalyst. The observed retention of the configuration was explained by the polyhydric cyclic structure of the carbohydrate [64]. The isomerization of methyl α-D-mannopyranoside and methyl β-D-galactopyranoside to corresponding D-gluco isomers was also found after several days of reaction.

**Figure 16:**
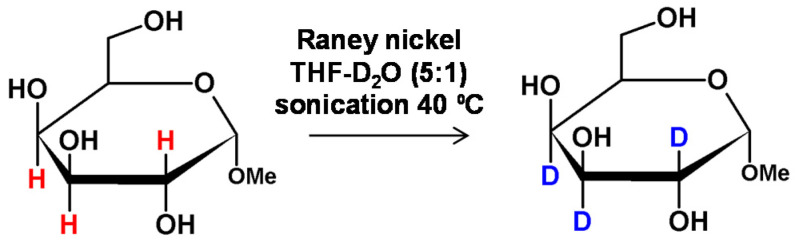
Methyl α-D-glucopyranoside deuteration catalyzed by Raney nickel under sonication as presented by Cioffi and co-workers [66].

As described by Cioffi and co-workers, the activation of Raney nickel by sonication allowed a microwave-assisted deuteration of nonreducing carbohydrates without racemization [65]. The 1-O-methyl-β-d-galactopyranoside, used as a model compound, was heated in 15-s intervals up to 36 times, using a microwave oven. It was found that the deuterium was incorporated without epimerization or compound decomposition. Further optimization of the Stuart’s method may be achieved by application of sonication which makes a higher level of deuterium incorporation possible (Figure 16) [66]. Microwave irradiation may also improve the deuteration however in this case the epimerization and substrate degradation was observed [65]. In the proposed solution deuteration was regioselective and occurred at the C2, C3 and C4 positions at the carbon atoms connected with hydroxyl group which was necessary for the HDX [67].

**Figure 17:**
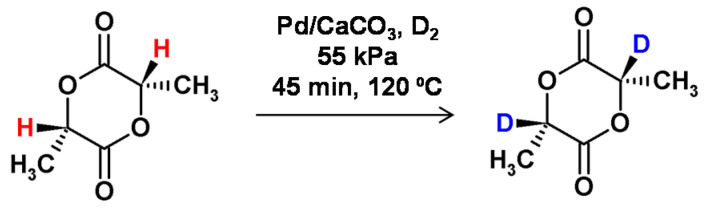
HSCIE method of selective H/D exchange at the carbon centers [68].

Vert and co-workers applied the HSCIE (high-temperature solid-state catalytic isotope exchange) technique to obtain the selectively deuterated lactide [68] and glycolide derivatives [69], which were applied as substrates for the synthesis of isotopically labeled biocompatible absorbable poly-α-hydroxy acids. It was found that the optimal reaction temperature was close to the melting point of the substrate. The hydrogen−deuterium exchange of L-lactide at 120 °C in the presence of the Pd/CaCO_3_ catalyst resulted in incomplete deuterium incorporation (Figure 17), but occurred without epimerization. Additionally, the reaction conditions were suitable for the tritium incorporation [70].

In 1990, Möbius and Schaaf [71] developed a method for the preparation of deuterated aliphatic hydrocarbons by metal-catalyzed HDX at higher temperatures (up to 290 °C). The reaction was performed in an autoclave, where a wire basket with the catalyst was placed above the substrate to be deuterated. The D_2_/D_2_O atmosphere was used to obtained deuterated derivatives under the pressure of around 25 Mpa. Under these conditions, water dissociates much more rapidly than at room temperature [72] and therefore Pd^0^ is able to insert oxidatively into the H-OH bond with the formation of a Pd(II) derivative [73].

**Figure 18:**
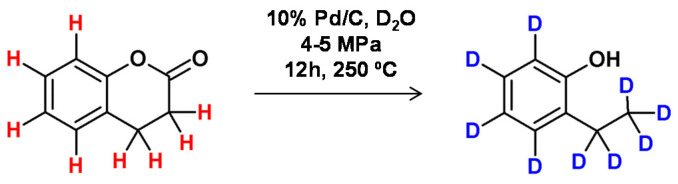
Lactone deuteration as presented by Matsubara et al. [74].

Based on the method developed by Matsubara and co-workers [74], complete deuteration of aromatic or aliphatic hydrocarbons was achieved by decarboxylation of carboxylic acids under hydrothermal reaction conditions. In this method, the model lactone molecule in D_2_O allowed the formation of the phenol derivative with a high yield in the presence of 10% Pd/C (5 mol %) at 250 °C and a pressure of 4–5 Mpa (Figure 18).

Previous studies by Sajiki and co-workers [75] on 5-phenylvaleric acid revealed the influence of reaction temperatures on the regioselectivity and deuteration. It was found that the benzylic hydrogen atoms were selectively exchanged to deuterons at room temperature, whereas at the higher temperature (160 °C), the deuteration in less reactive positions was also found, resulting in the formation of multideuterated derivatives. The proposed reaction conditions were compatible with compounds containing different functional groups in the form of carboxy, keto or hydroxyl groups, but the described reaction was characteristic for those with aryl-linked side chains. The proposed Pd/C–H_2_/D_2_O system may be also applied for the preparative formation of the phenylalanine selectively deuterated at the β carbon atom which takes place at 110 °C (6 h, 96% D) without racemization [76]. It was also found that at 160 °C the α position is also able for HDX, but these reaction conditions promote racemization (17% ee).

**Figure 19:**
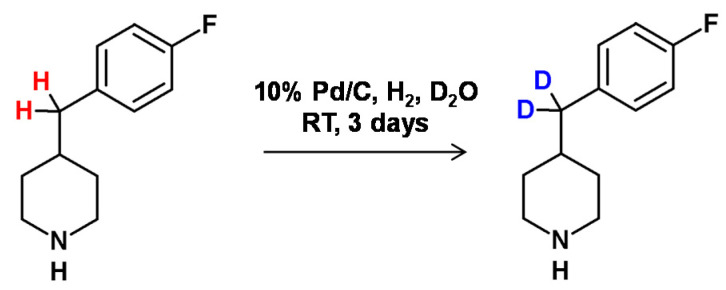
HDX reaction at the carbon center of piperidine derivative as presented by Proszenyák et al. [77].

In 2005, Proszenyák et al. [77] developed a method allowing higher efficiency of HDX for the benzylic hydrogen atoms of the piperidine derivative in the presence of Pd/C–H_2_–D_2_O, deuterated alcohols and DCl. Earlier, in 1986, Stock and Ofosu-Asante [78] presented a method for selective benzylic deuteration of the tetrahydronaphthalene carboxylic acid in the presence of a Pd/C catalyst under D_2_ atmosphere and deuterated acetic acid as the deuterating agent (Figure 19).

**Figure 20:**
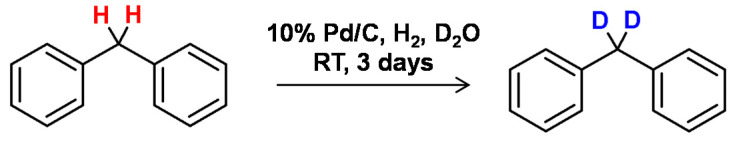
HDX at the carbon center presented by Sajiki et al. [79].

In 2005, Sajiki and co-workers [79] revealed that platinum catalysts present a higher tendency towards the deuteration of aromatic positions, whereas palladium catalysts prefer mostly aliphatic ones (Figure 20). Using this method, the efficient deuteration of phenol was obtained in the presence of 5% Pt/C at room temperature, whereas the palladium-catalyzed reaction needed a higher temperature (180 °C) to obtain the same level of deuteration.

Palladium and platinum catalysts may also be applied in a mixed catalyst system for the preparation of deuteration derivatives on the sterically hindered aromatic positions. It was found that the deuterium incorporation at the *ortho* position in 5-phenylvaleric acid in the presence of palladium (10% Pd/C) was only 14%, with platinum (5% Pt/C) 19%. In a mixed catalyst, the same reaction was characterized by almost complete deuteration (97% D) of the *ortho* position. Additionally, the synergistic effect of palladium and platinum complexes in stepwise deuteration was postulated as a useful tool in the case of a low degree of deuterium incorporation. [80].

HDX at the carbon center of alcohol molecules may also be catalyzed by molybdocenes. In this case, the reaction mechanism involves C–H bond activation by the metal catalyst. Deuteration may occur in different ranges, depending on the chemical structure of the alcohol used and can reach up to 99% in the case of benzylic hydrogens [81].

Most of the presented metal-catalyzed deuteration methods involve the application of second-row transition metals. In 2018, Prakash and co-workers developed a method of the regioselective deuteration of primary and secondary alcohols in the presence of first-row transition-metal catalysts [82]. In 2007 Hamid et al. presented the ‘borrowing hydrogen’ method of deuteration [83] which allows deuteration at the α and β carbon atoms of alcohols and amines by using a hydrogen-transfer catalyst. It was reported that manganese and iron pincer catalysts increase the deuterium incorporation in primary and secondary alcohols especially in the presence of a base. The presented mechanism of HDX involves the amido-complex formation with the used base which is a key factor for catalytic cycle initiation. The formed aldehyde is subsequently reduced by the deuterated metal complex formed by HDX on the catalyst mediated by D_2_O. This method was successfully applied in the deuteration on the α and β carbons of primary and secondary alcohols, as well as diols.

**Figure 21:**
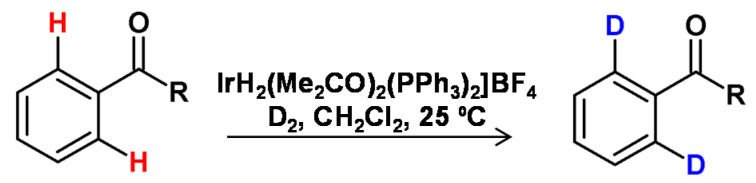
H/D exchange catalyzed by iridium catalyst as shown by Heys [85].

As presented by Bergman et al., the cationic iridium complexes may also activate C-H bonds [84], and therefore iridium-mediated HDX represents the largest number of published examples in the field of homogeneous metal catalysis. The most exploited area is the ortho-deuteration of aryl ketones and acetanilides. Starting with the investigations of the Heys [85] and Hesk [86] research groups, several different studies related to the effects of complex ligands [87], the deuterating agent [88], solvent [88,89,90], addition of bases [85] the amount of catalyst [36], the temperature, and the duration of the reaction [85] on the degree of deuteration and the substitution pattern in the substrate (Figure 21).

Further, Kröger et al. presented that the unsaturated carbonyl compounds were also suitable substrates for the above presented deuteration method reacting through a similar mechanism [88]. It was shown that β-hydrogen atoms underwent the H/D exchange with a good yield. It was also pointed out that the regioselectivity of the labeling depended on which deuterium source was used.

Buchanan et al., who developed catalysts for C-H bond activation [84] also presented the applicability of the soluble iridium complexes for the specific deuterium incorporation in aliphatic and nonfunctionalized aromatic substrates. A high degree of deuterium incorporation was obtained with hydrocarbons, alcohols, phenols, ethers, carboxylic acids, esters, and amides with D_2_O, [D6]acetone, or [D6]benzene [91].

**Figure 22:**
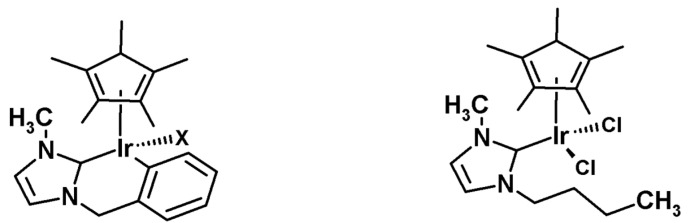
Iridium-carbene complexes presented by Peris et al. [92].

In 2006, Peris et al. demonstrated efficient HDX with diethyl ether, ethyl methyl ketone, isopropanol, and styrene with the *N*-heterocyclic iridium–carbene complexes in the presence of [D4]methanol (Figure 22) [92].

### 2.3. Metal-Catalyzed HDX Adjacent to Nitrogen Atom

**Figure 23:**
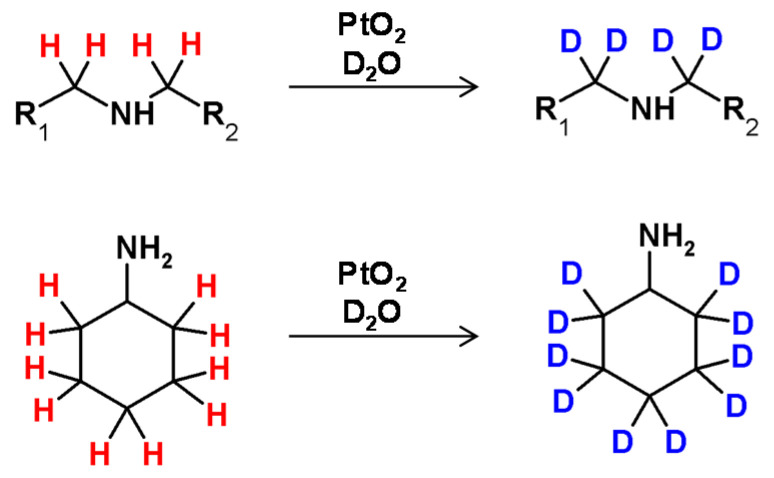
H/D exchange at carbon centers adjacent to nitrogen atom observed by Maeda et al. [93].

A first example of deuterium incorporation into the carbon centers in amines was reported in 1977, by Maeda et al. [93]. The hydrogen−deuterium exchange on the carbon atoms of primary and tertiary amines was performed in the presence of a deuterated form of Adam’s catalyst (platinum oxide treated under reductive conditions by D_2_ in D_2_O) (Figure 23). Such a solution allowed the selective deuteration at the β carbon of primary amines and at the α-C of the tertiary. The applied catalyst was activated by UV light or γ-radiation irradiation. PtO_2_, after activation with D_2_, was applied for the selective deuterium incorporation in nucleosides [94]. A strong dependency of the exchange selectivity upon the number and steric demand of the substituents on the nitrogen atom has been observed for the exchange of α-hydrogen atoms of aliphatic amines and amino acids with Adam’s catalyst (PtO_2_·H_2_O). It was found that the nitrogen atom bound to the surface of the applied catalyst. The efficiency of H/D exchange decreased in the following series tertiary > secondary > primary amines [93]. A first example of chiral carbon atom deuteration in amines with retention of configuration was presented by Jere et al. in 2003 [95]. In this case, the HDX of alanine or alaninol with complete retention was performed in the presence of ruthenium on carbon under D_2_ in D_2_O.

**Figure 24:**
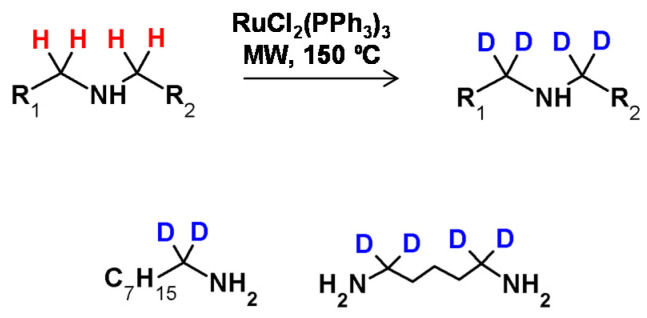
HDX at the carbon centers adjacent to nitrogen proposed by Matsubara and co-workers with selected examples [57].

The possibility of secondary amine deuteration via H/D exchange in the presence of RuCl_2_(PPh_3_)_3_ as the catalyst was investigated by Matsubara et al. [57]. It was found that the applied reaction conditions allowed selective deuteration at the α carbon atom with deuterium incorporations of up to 94% (Figure 24) The proposed reaction conditions were also applied in the deuteration of tertiary amines, resulting in 12% deuterium incorporation. As described, there was a possibility of selective amine deuteration at the α carbon atom under reaction conditions similar to those presented for alcohols. The configuration on the stereocenter in the β position depended on the temperature isotope exchange process and remained unaffected as long as the temperature did not exceed 100 °C [57].

**Figure 25:**
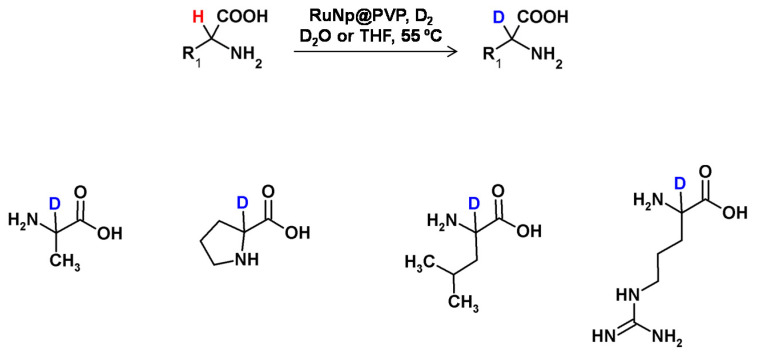
Schematic presentation of H/D exchange using ruthenium nanoparticles presented by Taglang et al. [96] with selected examples.

In 2015, Taglang and co-workers, reported that hydrogen−deuterium exchange at the chiral carbon centers of amino acid derivatives turned out to be stereoretentive (Figure 25) [96]. The authors proposed a mechanism of reaction in which the key step is the coordination of the nitrogen atom of the amino group to the surface of the applied catalyst which makes a ruthenium site and enables the C–H activation at the α carbon atom. A molecule thus-activated undergoes HDX, resulting in a selective α carbon deuterated derivative.

Alexakis et al. in 2005 analyzed different ruthenium(II) catalysts in the deuterium labeling reactions of piperidines, piperazines and several different dialkylamines in the presence of D_2_O as the deuterium source [97]. They found that while RuCl_2_(PPh_3_)_3_ was active for the deuteration of primary alcohols and amines, in the presence of RuCl_4_(CO)_6_ only secondary amine labeling was possible. The study on the deuteration of 4-benzylpiperidine in the presence of [Ru_2_Cl_4_(CO)_6_] as catalyst revealed the incorporation of an average of five deuterium atoms per molecule, however, the positions of the introduced deuterons were not precisely described [97].

The study of the increase of the deuterated yield led Roche’s group [98] to demonstrate that similar deuteration as shown by Taglang and co-workers [96] could be achieved in the presence of a Ru/C catalyst instead of Ru nanoparticles, H_2_ atmosphere in D_2_O and a base under mild temperature (70 °C) [99]. The presented methodology allowed the stereoretentive preparation of large quantities (up to 0.2 mols) of hydrosoluble amines with chiral centers, showing the applicability of this process for industrial application.

After successful deuteration of α, β carbons of alcohols in the presence of Ru-pincer complexes, Gunanathan and co-workers [100] presented the α deuteration of amines and amino acids by using D_2_O as deuterium source. The presented protocol is characterized by its selectivity for primary and secondary amines (no deuteration occurs at the α carbon of tertiary amines or alcohols). In the case of amino acids epimerization was observed.

**Figure 26:**
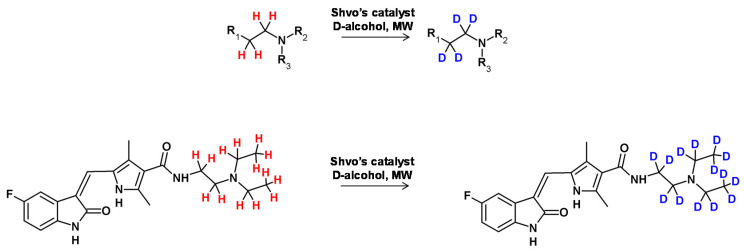
Schematic presentation of HDX in the presence of Shvo’s catalyst [102].

In 2012, Neubert et al. applied ‘borrowing hydrogen’ catalysis to perform the deuteration of tertiary amines [101]. In this study the Shvo catalyst was used to optimize the reaction conditions (Figure 26) [102]. The mechanism involved the formation of an intermediate in the form of iminium ion (in equilibrium with the corresponding enamine) mediated by the monomer of the catalyst bearing an available position in the coordination sphere of the metal atom. The reduced form of the applied catalyst underwent HDX in the presence of deuterating agent (D_2_O, deuterated alcohols) and subsequently transferred the deuterium atoms to the unsaturated bond of the enamine, generating a doubly labeled tertiary amine. It was found in the case of *N*,*N*,*N*-trihexylamine as the substrate that the deuterium incorporation was as high as 93% at the α and β positions in the presence of isopropanol-d8 as the solvent. This protocol was also successfully applied in the formation of deuterated pharmaceutical compounds, such as sunitinib (a kinase inhibitor).

**Figure 27:**
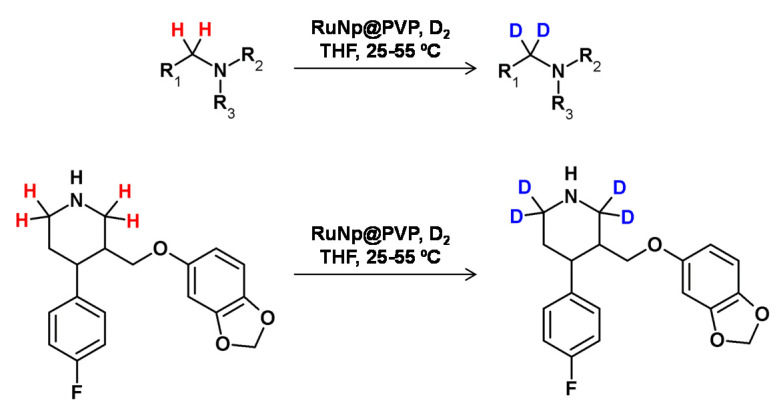
Ruthenium nanoparticles-catalyzed HDX according to Beller [101] and Pieters [103].

In 2014, Pieters et al. developed a method of regioselective α deuteration of nitrogen-containing bioactive compounds in the presence of ruthenium nanoparticles supported on polyvinylpyrrolidone under the D_2_ atmosphere [103] (Figure 27). Using this method, the authors obtained high deuterium incorporation with complete regioselectivity.

**Figure 28:**
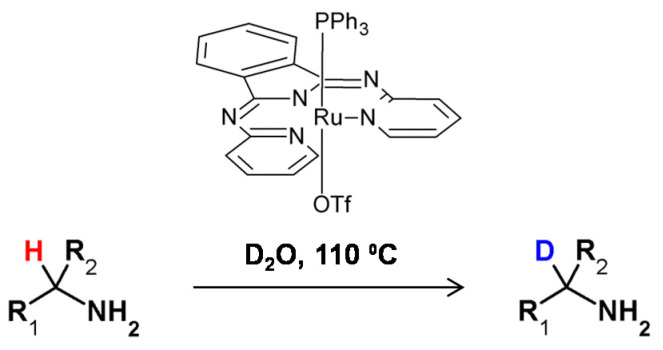
Model metal-catalyzed HDX reactions presented by Szymczak et al. [104].

In 2016, Hale and Szymczak proposed a method of stereoretentive deuteration in the presence of Ru-bMepi complex that avoided the use of D_2_. In this method the complete stereoretention in the case of a chiral amine was observed. However, in the case of chiral alcohol deuteration, complete racemization occurred (Figure 28) [104].

In 2016, Jackson et al. [105] developed a method of electrocatalytic deuteration of amines and alcohols which overcame the problem of the D_2_ solubility in an aqueous environment, by in situ generation of D_2_. Using this method, different deuterated derivatives were prepared with high level of deuterium incorporation, but with low yield.

A main challenge in this field was the development a stereoretentive hydrogen−deuterium exchange at the chiral carbon centers. Although the Rousseau reaction can be considered as stereospecific, an example of deuteration at the chiral carbon atom was presented in his paper. Maeda et al. also investigated the stereospecificity of the HDX in amines and found that at temperatures above 100 °C, L-alanine started to racemize [94]. During the analysis of selective HDX at the β carbon of phenylalanine in the presence of Pd/C catalyst under H_2_, the additional deuteration at the α carbon was observed at 160°C, with partial racemization of the chiral carbon atom.

Pyrimidine bases, including uracil or cytosine, may also be deuterated in the 5- and 6-positions in the presence of a Pd/C–H_2_/D_2_O mixture at 110 °C [106]. The deuterium was incorporated in the 5-methyl group of thymine in addition to the 6-position, and no side products have been reported. Purine nucleosides, including adenosine or inosine, were successfully and chemoselectively deuterated in the 2- and 8-positions [107]. The lower HDX levels were noted for the pyrimidine bases and analyzed nucleoside in the presence of CD_3_OD as a solvent and deuterating agent in the form of D_2_O.

In 2001, Hardacre and co-workers reported the application of a catalyst activated by hydrogen reduction in the preparation of deuterated imidazoles and imidazole salts. The substrate solution in D_2_O was treated by the reaction mixture, degassed by several cooling/thawing cycles [108].

**Figure 29:**
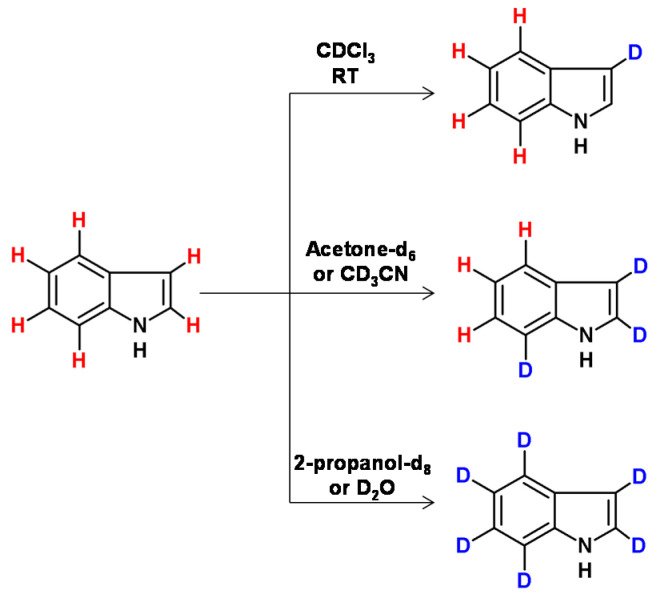
H/D exchange described by Gavrisch Yau and Gawrisch [109].

Raney nickel catalyst in the presence of [D6]acetone, [D3]acetonitrile, [D1]chloroform, D_2_O or [D8] 2-propanol allowed the selective deuteration of specific positions in tryptophan derivatives, based on their nucleophilicity [109]. The influence of the differing nucleophilic potential of these positions and the indole ring orientation on the catalyst surface influenced by the solvent were responsible for the selectivity of deuteration (Figure 29). In the case of deuteration catalyzed by Raney nickel, it was found that only the hydrogen atoms at the α-carbons underwent selective deuteration in quinuclidine at higher temperatures, a longer reaction time and in the presence of D_2_O (100 °C, 40 h, 2 reaction cycles; ≥99.7% D). It was also reported that less than 1% of deuterium was incorporated into the β and γ carbon centers [110].

Previously Hickey et al. reported the extended spectrum of the substrate to aniline and benzylamine derivatives [111]. The application of [Ir(cod)(acac-F6)] (acac-F6=hexafluoroacetylacetonate) and D_2_ allowed *ortho* HDX, relative to the position of the amino or methylamino group.

In 2017, Loh et al. [112] proposed a new deuteration strategy, based on the application of photoredox deuteration and tritiation. The method was successfully applied in the formation of isotopically labeled derivatives of pharmaceuticals containing alkyl amine moieties. The developed method involved the formation of an α-amino radical via a single-electron transfer occurred in the presence of an iridium(III) catalyst, previously excited by visible blue light. The application of a hydrogen atom transfer (HAT) catalyst allowed the abstraction of deuterium (or tritium) to form labeled derivatives. It was also found that application of thiols as HAT catalysts was crucial for the preparation of labeled compounds. This process was optimized and then applied in the preparation of gram scale products. All of the reported examples met all the requirement characteristic for internal standards which may be applied in quantitative analysis by mass spectrometry (more than 4 D and less than 0.1% of the unlabeled compound). Moreover, the proposed process may be performed in the presence of several functional groups, presenting a high selectivity towards exchange at the C(sp^3^)–H centers adjacent to the nitrogen atom and retention of the stereochemistry, even when HDX proceeds at the chiral center. In 2015, Hu and co-workers described an example of deuteration based on the single-electron transfer mechanism [113]. In the presented process, deuteration took place only at the α carbon atom of the amine when the radical was stabilized at a benzylic position. It was also found that the presence of the nitrogen was not required for the deuterium incorporation.

### 2.4. pH Dependent and Metal-Catalyzed HDX Adjacent to Sulfur Atom

Sulfur-directed deuteration is not so common in scientific literature. Only a few papers report deuteration at the α carbon position of sulfur center. In 2018, the application of ruthenium on carbon under D_2_ in the H/D exchange in thioesters was presented by Gao et al. [114] By using this method a number of deuterium-labeled drugs or amino acids and peptides have been successfully prepared with a good regioselectivity towards the α carbon to the sulfur center. Additionally, in the case of chiral molecules involved in this process, the retention of the configuration was found. It was also suggested that the mechanism of the described process was the same as that proposed for the ruthenium nanoparticle-mediated deuteration of amino acids [96].

**Figure 30:**
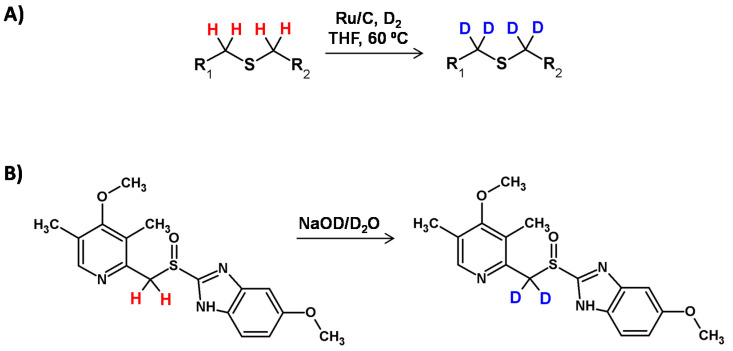
Schematic presentation of H/D exchange at the carbon center connected with the sulfur atom presented by Gao et al. [114] (**A**) and Rauk et al. [116] (**B**).

A great review presenting the possibility of pH dependent and metal-catalyzed hydrogen−deuterium exchange adjacent to the sulfur atom was presented by Michelotti and co-workers [115]. Rauk et al. [116] demonstrated a deuteration process in the presence of NaOD in D_2_O or just in D_2_O for substances containing the methylsulfonyl or methylsulfinyl groups. They reported that sulfonyl (-SO_2_-) and sulfinyl (-SO-) groups should present similar conjugative ability with adjacent carbanionic centers (Figure 30). It was also presented that -SO- group was much less effective than a -SO_2_- group in promoting deuteration at the α carbon center. The previously reported HDX of dimethyl sulfoxide in the presence of deuteroxide may have resulted from the enhanced reactivity of applied bases in dimethyl sulfoxide solution. Additionally, an unexpected difference in the deuteration level at the two methylene hydrogens was found [101].

The possibility of complete deuteration of methylene hydrogens adjacent to the sulfinyl group was presented by Redondo and co-workers [117]. The reaction proceeded at room temperature when the sodium salts of compounds containing [(pyridylmethyl)sulfinyl]benzimidazole structural core were dissolved in a solvent serving as a deuterium source, including D_2_O and CD_3_OD. The presented process resulted from the weak acidity of the methylene hydrogen atoms, and was also observed in a nondeuterating solvent like DMSO-d_6_ in the presence of aa catalytic amount of NaOH. The described HDX was monitored by ^1^H NMR which also revealed the stereoselectivity of deuterium incorporation [116,118].

Transition metal-catalyzed H/D exchange can be classified into three categories. The first one involves the C−H bond activation which generates an organometallic intermediate, bearing a metal−carbon bond as a result of the carbon−hydrogen bond activation. The second one is denoted as C−H insertion catalysis as a key working mode, whereas the third one is related to the photoredox catalysis [119].

The advantages of metal catalyzed hydrogen−deuterium exchange processes are that they are usually easy to perform and most of the presented catalysts are commercially available. The limitation of the presented methods is the high price of the catalyst, necessity of compound purification, long reaction times or the necessity for the application of high pressure.

## 3. Conclusions

The presented manuscript provides an overview of the methods of H/D exchange reactions at the carbon centers. From the first reports of HDX at the carbon center in alcohols and amines, the state-of-the-art in this field has expanded enormously, mostly due to the application of metal catalysis. proving its validity and utility. Nowadays, the deuterated compounds play a crucial role as the internal standards in the quantitative analysis by the LC-MS technique. Although the possibilities of deuterated standards in quantitative investigation of compounds are beyond doubt, their application in medical diagnosis may be limited, due to the high costs of their preparation. Therefore, despite significant progress there is still a strong need for the development of new, simple, rapid and cost-efficient methods of carbon deuteration. It may be speculated that the new techniques of hydrogen−deuterium exchange at the carbon centers will be developed in the near future. It may be expected that they will be characterized by a low price of preparation, the stability of the introduced deuterons, long-term storage of the deuterated derivatives at room temperature, an appropriate mass difference for MS quantification, stability of introduced deuterons during chromatographic separation and co-elution with nondeuterated isotopologues. It may be expected that further work in the presented area will be forthcoming.

## Data Availability

Not applicable.
